# Paired miRNA- and messenger RNA-sequencing identifies novel miRNA-mRNA interactions in multiple myeloma

**DOI:** 10.1038/s41598-022-16448-0

**Published:** 2022-07-15

**Authors:** Kristin Roseth Aass, Tonje Marie Vikene Nedal, Synne Stokke Tryggestad, Einar Haukås, Tobias S. Slørdahl, Anders Waage, Therese Standal, Robin Mjelle

**Affiliations:** 1 Department of Clinical and Molecular Medicine, Centre of Molecular Inflammation Research, Norwegian University of Science and Technology, Gastrosenteret, Prinsesse Kristinas gt. 1, 7491 Trondheim, Norway; 2 Department of Clinical and Molecular Medicine, Norwegian University of Science and Technology, Erling Skjalgssons gt. 1, 7491 Trondheim, Norway; 3 Department of Hematology, Stavanger University Hospital, 4011 Stavanger, Norway; 4 Department of Hematology, St. Olavs University Hospital, 7030 Trondheim, Norway; 5 Bioinformatics Core Facility - BioCore, Norwegian University of Science and Technology NTNU, 7491 Trondheim, Norway

**Keywords:** Myeloma, miRNAs

## Abstract

Multiple myeloma (MM) is an incurable cancer of terminally differentiated plasma cells that proliferate in the bone marrow. miRNAs are promising biomarkers for risk stratification in MM and several miRNAs are shown to have a function in disease pathogenesis. However, to date, surprisingly few miRNA-mRNA interactions have been described for and functionally validated in MM. In this study, we performed miRNA-seq and mRNA-seq on CD138 + cells isolated from bone marrow aspirates of 86 MM patients to identify novel interactions between sRNAs and mRNAs. We detected 9.8% significantly correlated miRNA-mRNA pairs of which 5.17% were positively correlated and 4.65% were negatively correlated. We found that miRNA-mRNA pairs that were predicted by in silico target-prediction algorithms were more negatively correlated than non-target pairs, indicating functional miRNA targeting and that correlation between miRNAs and mRNAs from patients can be used to identify miRNA-targets. mRNAs for negatively correlated miRNA-mRNA target pairs were associated with gene ontology terms such as autophagy, protein degradation and endoplasmic stress response, reflecting important processes in MM. Targets for two specific miRNAs, miR-125b-5p and miR-365b-3p, were functionally validated in MM cell line transfection experiments followed by RNA-sequencing and qPCR. In summary, we identified functional miRNA-mRNA target pairs by correlating miRNA and mRNA data from primary MM cells. We identified several target pairs that are of potential interest for further studies. The data presented here may serve as a hypothesis-generating knowledge base for other researchers in the miRNA/MM field. We also provide an interactive web application that can be used to exploit the miRNA-target interactions as well as clinical parameters associated to these target-pairs.

## Introduction

MicroRNAs (miRNAs), about 22 nucleotides in length, are among the most studied groups of small RNAs, and repress gene expression by RNA silencing^1^. miRNAs are predicted to regulate most of the transcribed genes in the genome by binding to the 3′ untranslated regions (3′UTRs)^2^. The effect of a miRNA on a given mRNA can be difficult to measure as most mRNAs are targeted by multiple miRNAs, and the expression of miRNAs vary under different physiological conditions. The key to understanding miRNA function is to identify target mRNAs. Traditionally, potential targets have been identified using in silico prediction algorithms or by over-expressing miRNAs in cell lines. The latter has the disadvantage of saturating the miRNA machinery leading to off-target effect and disruption of the natural miRNA targeting within a cell^3^. Another approach is to perform paired miRNA-mRNA profiling on the same samples and identify negatively correlated miRNA-mRNA pairs^4,5^. The advantage of this approach is that all expressed miRNAs in the cell can be investigated at the same time and that in vivo miRNA-target interaction can be identified.

MM is a B-cell malignancy characterized by clonal expansion of malignant plasma cells in the bone marrow. Altered miRNA expression is shown to affect key biological processes in MM, including apoptosis and proliferation^6–11^. However, to date, still relatively few miRNA-mRNA interactions have been described in MM^12^. Furthermore, studies often focus on single miRNA-mRNA interactions, ignoring that regulation of a complex repertoire of mRNAs by the same miRNA may be causing the observed effect. This concept is crucial as the same miRNA may act as both tumor suppressor and oncogene by regulating genes with opposing effects on disease pathogenesis^12^. It is therefore important to identify all mRNA targets of miRNA in an in vivo setting before miRNAs can be considered as treatment targets.

To characterize global miRNA-target interactions in MM we performed miRNA-seq and mRNA-seq on RNA isolated from purified bone marrow plasma cells from 86 patients at diagnosis. No such comprehensive paired miRNA-mRNA sequencing experiment has been conducted in MM. We demonstrated that functional miRNA target interactions can be identified by correlating miRNA and mRNA expression in the cancer cells from each patient. The results from this study are available through an interactive web application that can be utilized to investigate miRNA-target interactions.

## Results

### Study design and overview of small RNA- and mRNA-sequencing data

**Figure 1:**
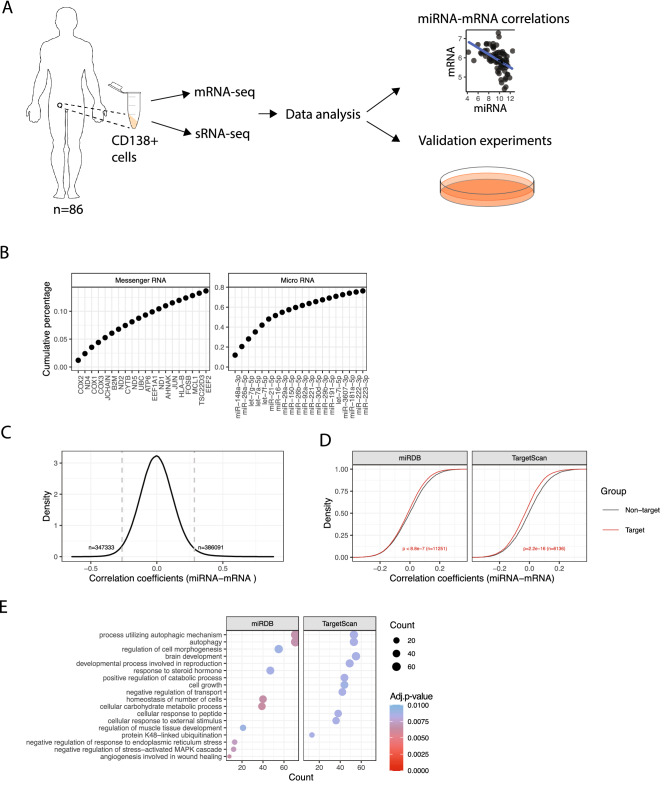
(**A**) Study design and analysis overview (**B**) Cumulative expression of the top 20 highest expressed mRNAs (left) and miRNAs (right). (**C**) Density plot showing the distribution of correlation coefficients for all miRNA-mRNA Pearson’s correlations. Indicated is the number of significant positive correlations (n = 386,091) and significant negative correlations (n = 347,333). (**D**) Cumulative distribution of miRNA-mRNA correlation coefficients for in silico predicted miRNA targets and non-targets. Shown are targets predicted by miRDB and TargetScan. The p-values represent the difference between the target and non-target groups and the numbers indicate the number of predicted miRNA-target pairs. (**E**) Gene ontology analysis of the genes with most significant miRNA-mRNA correlations and the best *in silio* prediction score for miRDB and TargetScan and (n = 1327 and 874 for miRDB and TargetScan targets, respectively) (see Methods). The color of the dots and the color legend “Adj.*p*-value” indicate the Benjamini–hochberg adjusted *p*-value. The size of the dots and the legend “Count” indicate the gene ratio (see Methods). Shown is the gene ontology for biological processes.

Paired small RNA- and mRNA-seq was performed on CD138 + cells isolated from bone marrow aspirates of 86 MM patients (Fig. 1A). On average, 54 million reads were mapped to the human genome (Additional file 1, Fig. S1A). We detected several types of small RNAs in our samples (Additional file 1, Fig. S1B), but in this study we chose to focus on the miRNA fraction (Additional file 1, Fig. S1C), A total of 1757 unique miRNAs were detected and of those, 161 were expressed with at least 1 count per million (cpm) in all 86 samples. The most highly expressed miRNA was miR-148a-3p. Six miRNAs contributed with about 50% of the reads in the libraries (Fig. 1B). Messenger RNA-seq was performed on the same RNA from the same samples. On average 15 million reads mapped to the human genome. We detected 6116 mRNAs with an expression above 1 cpm in all samples. The most highly expressed mRNAs were genes coding for the proteins cytochrome c oxidase, NADH dehydrogenase and the prognostic marker B2M (Fig. 1B).

### General correlations between miRNA and mRNA expression in patient samples

By sequencing miRNAs and mRNAs from the same samples, we have a unique possibility to investigate the correlation between the two RNA classes, and how miRNA and mRNAs interact to regulate each other. To investigate interactions between miRNAs and mRNAs we first calculated the Pearson's correlation coefficients for all possible miRNA-mRNA pairs in all patient samples irrespectively of predicted miRNA targets. We detected 733 424 significantly correlated miRNA-mRNA pairs (9.8% of all possible pairs) of which 5.17% were positively correlated and 4.65% were negatively correlated (Fig. 1C). The mean Pearson correlation coefficients for the significantly positively and negatively correlated pairs were 0.288 and −0.260, respectively. Next, we investigated if the abovementioned significantly correlated miRNA-mRNA pairs were enriched for MM-dysregulated genes. We utilized a recent meta-analysis in MM with a list of 1362 MM-dysregulated genes^13^. We found that the top 10% strongest positive correlation were enriched for MM-dysregulated genes (293 MM-dysregulated genes detected) both compared to genes with the top 10% negative correlations and compared to a random selection of genes (*p* < 2.2e−16 and *p* = 2.698e−08, respectively, Chi-squared test), (Supplementary Table 1). The top 10% negatively correlated genes did not show any enrichment for MM dysregulated genes (106 MM-dysregulated genes detected) (Supplementary Table 1).

MiRNAs that are located within protein coding genes tend to be transcribed together with their host genes and have similar expression profile^4,14^, which could explain why some miRNA-mRNAs pairs are positively correlated. To investigate this, we grouped the miRNAs into two groups, intergenic and intragenic miRNAs, and correlated the mRNA and miRNA expression profiles within the two groups. The intragenic miRNAs were significantly more positively correlated compared to the intergenic miRNAs, indicating that intragenic miRNAs are co-expressed with their host genes (Additional file 1, Fig. S2A, B). This mechanism explains some of the positive correlation in our data. We also observed some positively correlated intergenic miRNAs and mRNAs, which point towards other regulatory mechanisms than co-transcription (Additional file 1, Fig. S2C).

### MiRNAs and predicted target gene correlations

To look closer into the more relevant correlations we calculated the Pearson’s correlation coefficients for miRNAs and the predicted target genes. We hypothesized that some of the negative correlations could be due to miRNA targeting mechanisms. To detect potential miRNA-target interactions we performed in silico target prediction using two prediction tools, TargetScan^15^ and miRDB^16^. We detected 31,461 common miRNA-target pairs between TargetScan and miRDB after removing lowly expressed target genes and target genes with low prediction score (see Methods). Next, we compared the distribution of correlation coefficients between miRNA-target and -non-target pairs. We found that predicted miRNA-target pairs were significantly more negatively correlated compared to miRNA-non-target pairs, using predictions from both TargetScan and miRDB (Fig. 1D). This indicates down-regulation of predicted miRNA targets in patient samples and shows that functional miRNA-target interaction can potentially be identified from paired miRNA-mRNA patient data.

Next, we performed gene ontology (GO) analysis on the miRNA-mRNA target pairs that were negatively correlated in the patient data and had the best prediction score to investigate if the high probability targeted genes were involved in similar biological processes. The GO analysis revealed that the negatively correlated, predicted target genes were significantly enriched in biological processes important in MM cells, such as autophagy, protein degradation and endoplasmic stress response (Fig. 1E). Of note, the GO term autophagy was overrepresented by predictions from both miRDB and TargetScan (Fig. 1E).

### Effects of miR-125b-5p and miR-365b-3p on gene expression in MM cells

**Figure 2:**
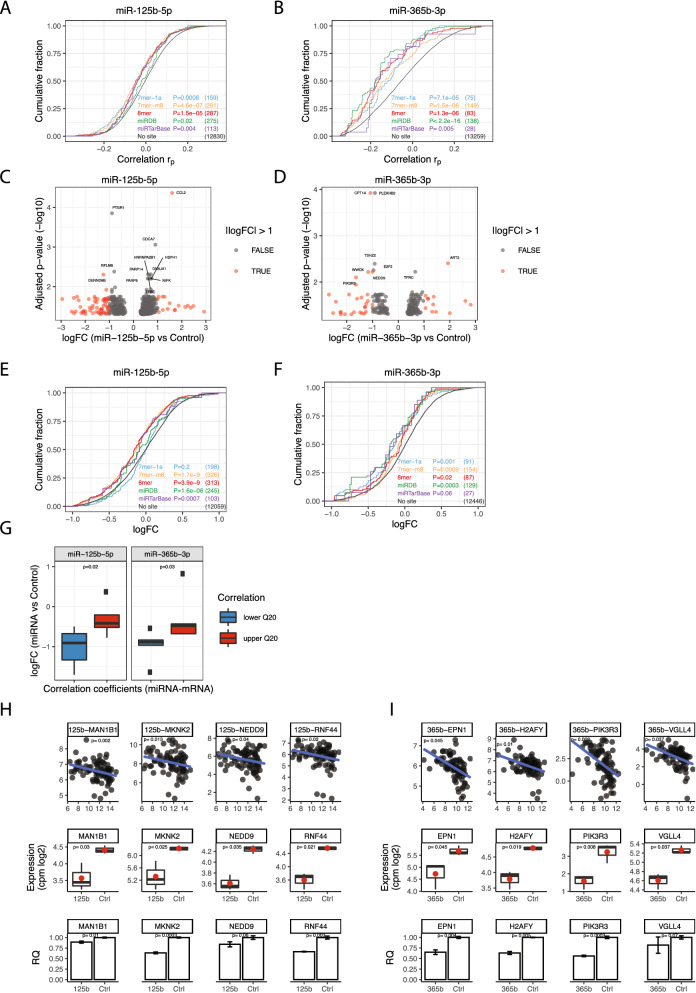
Identifying mRNA targets of miRNA-125b-5p and miR-365b-3p in patient’s data and INA-6 cells. (**A**) Cumulative distributions of miRNA-target correlation coefficients for miRNA-125b-5p and (**B**) miR-365b-3p in the patient samples. The colours represents mRNA-targets of different type as predicted by miRDB and TargetScan and miRTarBase. Differences in correlation coefficients between mRNAs with and without predicted target sites were tested (P-values from one-sided Kolmogorov–Smirnov test). The number of mRNAs analyzed in each group is listed in parentheses. (**C**) Significantly differentially expressed mRNAs (adjusted *p*-value < 0.05) upon miR-125b-5p or (**D**) miR-365b-3p overexpression in INA-6 cells. The volcano plot shows the log2FC-values on the X-axis and the inverse Benjamini-hochberg-adjusted p-values on the y-axis. Messenger RNAs with absolute log2FC above 1 is shown in red. The most significant mRNAs (−log10 *p*-value > 2) are indicated with gene-name. (**E**) Cumulative distributions of miRNA-target correlation coefficients after overexpressing miR-125b-5p or (**F**) miR-365b-3p in INA-6 cells. See A) for explanations of the plot. (**G**) Comparison of patient’s correlation coefficients and logFC values from the transfection experiments. The groups “lower Q20” and “higher Q20” are the patient’s correlation coefficients for the significant mRNAs from the transfection experiment, grouped into the upper and lower Q20 quantiles, such that “lower Q20” is the Q20 most negative coefficients and “higher Q20” is the Q20 most positive coefficients. The p-values are calculated using a two-sample one-sided student’s t-test in R. (**H**) Pearson correlation coefficients between miRNA and target gene expression (top panel), RNA-seq data (middle panel) and gene expression evaluated by RT-qPCR (lower panel) for the top four miRNA-target pairs for miRNA-125b-5p or (**I**) miR-365b-3p that were consistent both in the patient’s data and in the transfection experiment. The pairs were chosen by first selecting the most negatively correlated pairs from the patient’s data, then among those, selecting the most significant pairs from the transfection experiment. The *p*-values in the middle (RNA-seq) and lower (RT-qPCR) panels are calculated using a two-sample one-sided student’s t-test in R. The *p*-values for the miRNA-mRNA correlation (upper panel) was calculated using the cor.mtest within the corrplot (v0.84) package in R.

The miRNA miR-125b-5p was one of the most highly expressed miRNAs in our dataset and has previously been implicated in MM^17^. miR-365b-3p was the miRNA with most significant negative correlation with mRNAs in the patient data and belongs to the miR-193b-365-cluster of miRNAs that has previously been implicated in MM^18^. We therefore set to further investigate the putative function of these two miRNAs by validating their predicted mRNA targets. First, we investigated the correlations between the two miRNAs and the predicted targets in the patient samples. Targets of miRNA can be predicted based on Watson–Crick complementarity of the mRNA with the miRNA seed sequence (2–8 nucleotides starting from the 5 ´end. The longer sequence at the mRNA 3´UTR with matching nucleotides the more likely it is to be targeted^19^. We grouped the TargetScan targets into three groups, 8-mer, 7mer-m8 and 7mer-a1, as defined by the TargetScan algorithm^19^, as well as including miRDB predicted targets and previously validated targets (miRTarBase). We found that targets of miR-125b-5p tended to be significantly more negatively correlated to miR-125b-5p than non-target genes (Fig. 2A). This was true for all types of TargetScan targets as well as miRDB predicted targets and miRTarBase. For miR-365b-3p, we also observed a similar shift towards negative correlations for target genes compared to non-target genes and the strongest negative correlation was observed for 8mer targets, which is also the strongest target site type (Fig. 2B). Together, these results show that in silico predicted miRNA targets tend to be more negatively correlated compared to mRNA without predicted target sites, indicating in vivo miRNA-target interactions.

To further identify miR-125b-5p and miR-365b-3p target genes, we transfected the MM cell line INA-6 with miR-125b-5p and miR-365b-3p mimics and performed RNA-seq to measure the effect of the miRNAs on mRNA expression (Fig. S3). We detected 779 differentially expressed transcripts (5.4%) when comparing the miR-125b-5p transfection to the negative control transfection, of which 505 transcripts were up-regulated and 264 were down-regulated (Supplementary Table 2, Fig. 2C). For miR-365b-3p we detected 299 differentially expressed transcripts (2.1%) when comparing the miR-365b-3p transfection to the negative control transfection, of which 189 transcripts were up-regulated and 110 were down-regulated (Supplementary Table 3, Fig. 2D). For miR-125b-5p, when only considering transcripts with an absolute logFC values above 1, 69 transcripts were down-regulated and 20 were up-regulated, indicating an overrepresentation of down-regulated transcripts upon miR-125b-5p transfection (*p* = 4.4e−6, Chi-squared test) (Fig. 2C). For miR-365b-3p, 32 transcripts were significantly down-regulated more than 1 logFC and 14 up-regulated more than 1 logFC, also indicating an overrepresentation of down-regulated transcripts upon miR-365b-3p transfection (*p* = 0.01, Chi-squared test) (Fig. 2D).

Next, we investigated how miRDB and TargetScan-predicted transcripts were affected in the transfection experiment. We grouped the transcripts into the same abovementioned target-groups. For miR-125b-5p, we found that miRDB predicted transcripts, miRTarBase and TargetScan predicted transcripts with site-types 8-mer and 7mer-m8 were significantly down-regulated compared to transcripts without target sites, in agreement with these site-types being the strongest site-types (Fig. 2E). For the site-type 7mer-1a we observed no significant difference compared to transcripts without target sites. For miR-365b-3p, both miRDB-predicted transcripts, miRTarBase and transcripts within all three TargetScan-predicted target groups were significantly more down-regulated compared to transcripts without predicted target sites (Fig. 2F). Finally, we investigated the concordance between the transfection experiments and the miRNA-mRNA correlation coefficients in the patient samples. First, we identified the mRNAs with significantly altered expression in the transfection experiment that were also predicted targets for miR-125b-5p and miR-365b-3p. Then we grouped the mRNAs into two groups based on the correlation coefficients with the miRNAs in the patient samples. We found that target mRNAs that had the most negative correlations coefficients (lower 20th quantile) with the miRNA tended to be more downregulated in the transfection experiment than mRNAs with higher correlation coefficients (upper 20th quantile) (Fig. 2G). Thus, the transfection experiments are in concordance with the observations from the patient data.

To validate the RNA-seq results we performed RT-qPCR on the top four genes for miR-125b-5p and miR-365b-3p that showed significant down-regulation in the RNA-seq and that were most negatively correlated in the patient data. INA-6 MM cells were transfected with miRNA mimics for the two miRNAs and negative control miRNA. Six of the eight tested genes were significantly down-regulated in the mimic-transfected samples compared to negative control samples, including MAN1B1, MKNK2, RNF44 for miR-125b-5p and EPN1, H2AFY, PIK3R3 for miR-265b-3p (Fig. 2H, I). The two last genes (NEDD9 and VGLL4) were also down-regulated in the RT-qPCR experiment, but not significant.

## Discussion

In this study, we performed paired miRNA-seq and mRNA-seq on CD138 + cells collected at diagnosis from 86 MM patients. This is the largest profiling of sRNAs in MM and the first study to perform paired miRNA and mRNA profiling by sequencing in MM. The major advantage of our study is that we have performed paired miRNA and mRNA profiling from the same patients, analyzed from the same RNA samples. This enables investigation of miRNA-target interactions in an in vivo setting. Paired miRNA and mRNA analysis have previously been performed in MM^20^, however, this study had fewer samples, was not sequencing-based, and miRNA-target interactions were not analyzed in detail.

Using two different target prediction tools, miRDB and TargetScan, we showed that miRNA-mRNA target pairs tended to be more negatively correlated than miRNA-mRNA pairs for which no target interaction was predicted, implicating that the correlation of mRNA and miRNA in primary MM cells can indeed detect functional target-pairs. There are several advantages of using this approach to investigate miRNA activity in cancer patient material. First, novel miRNA-target interactions have traditionally been identified by over-expressing a particular miRNA followed by mRNA-profiling to look for altered mRNA expression. The disadvantage of this approach is that the miRNA of interest is expressed at a much higher level than what is physiologically relevant for a cell. This can lead to off-target effects, saturation of the miRNA biogenesis machinery, and false interactions to be identified^3^. Using a correlation approach, miRNA-target interactions can be identified without over-expressing a particular miRNA, and we are not limited to investigating only a specific miRNA. Second, over-expression experiments do not properly reflect the in vivo miRNA targeting activity, and is not easily conducted on patient cells, which is the reason why human miRNA-target interactions have been identified and validated using human cell lines, often easy-to-transfect cell lines as HEK293T. Here, we show that by correlating the expression of miRNAs and mRNAs, novel target interactions can be identified, demonstrated by a strong negative shift of correlation coefficients for in silico predicted miRNA-mRNA pairs. However, it should be noted that the in silico predictions from TargetScan and miRDB are not functionally validated and miRNAs could affect mRNAs indirectly by for instance altering transcription factors that regulate multiple mRNAs. Direct validation methods such as luciferase reporter assays should be applied to ensure that the miRNA-mRNA pairs are true interactions. miRTarBase contains several such validated targets and were included in the current study to add to the in silico predictions.

Another finding in the study was that genes that were strongly positively correlated with miRNA were enriched for genes frequently dys-regulated in MM. This could be related to the observation that genes with intragenic miRNAs are more positively correlated with miRNAs that genes with intergenic miRNAs (Fig. S2A), although we only found 18 intragenic genes among the 1327 dys-regulated MM genes, indicating that this effect is likely only minor. However, previous studies have found correlative behavior between miRNA and cancer-specific genes^21^. A Pan-cancer analysis on miRNA-mRNA correlations showed that the top-ranked positive correlations are significantly involved in processes related to immune cell differentiation and cell membrane signaling^22^. This study concluded that strong miRNA-gene correlations in cancer are likely explained by factors such as (1) miRNA inhibition of the upstream suppressor of the gene; (2) co-transcription by shared transcription factors; (3) super-enhancer mediated miRNA-gene co-expression, and (4) direct binding of miRNA to the regulatory regions of the partner gene^22^.

Interestingly, the genes predicted as targets for the miRNAs were involved in cellular processes such as handling of endoplasmic reticulum (ER)-stress and autophagy, processes that are implicated in proteasome inhibitor drug resistance in MM^23–26^. However, we also detect GO terms not directly related to MM, such as development and morphogenesis. Developmental genes are known to be enriched for miRNA targets^27^ which could explain why these GO terms are identified as significant.

To further validate that the correlation approach is identifying functional miRNA-mRNA target pairs, we selected two of the miRNAs, miR-125b-5p and miR-365b-3p, for an in vitro transfection- RNA-seq experiment. We found that for both miRNA candidates, mRNAs with predicted target sites tended to be down-regulated compared to mRNAs without predicted target sites. A further support to the use of correlation coefficients in identifying functional miRNA targets came from the good concordance between the logFC values and the correlation coefficients, meaning that target genes detected as down-regulated in the transfection experiment were also negatively correlated with its corresponding miRNA in the patients’ data. The four most downregulated genes at the RNA-seq that also were most negatively correlated in the patient data was finally confirmed by miR-125b and 365b transfection followed by qPCR. One of the targets for miR-125b was MKNK2, which encodes MAP kinase-interacting kinase (MNK2), a kinase regulating cap-dependent translation via phosphorylation of eIF-4E^28^. In MM, MNK2 was shown to facilitate selective translation of proteins necessary for MM proliferation and ER-stress response^29,30^. Supporting our correlation-based identification of MKNK2 as a miR-125b target, others have also reported the same interaction. A recent study found a negative correlation between miR125b and MKNK2 in ovarian cancer tissue and further showed that miR-125 targeting of MKNK2 was promoting autophagy in chemo-resistant cancer cells^31^. MiR-125b targeting of MKNK2, and MAN1B1, another validated target in our qPCR experiment, was also demonstrated in breast cancer^32^. Furthermore, miR-125b-MAN1B1 target activity was shown in hepatocellular carcinoma^33^. NEDD9 was significantly downregulated in our transfection-RNA-seq experiment, while not within threshold for significance in our qPCR experiment. It was however previously shown to be a direct target of miR-125b in melanoma promoting increased invasion and metastasis^34^. RNF44 was identified as a target of miR-125b in pancreatic beta cells^35^.

For miR-365b, which is a less studied miRNA, the interactions identified in this study has to our knowledge not been demonstrated before. However, as we found literature-supported miR-125b targets, the miR-365b target pairs are also likely biologically relevant. The genes validated as targets of miR-365b included EPN1, H2AFY, PI3KR3 and VGLL4, in which all have been reported as players in tumor biology^36,37^, and interestingly both VGLL4 and EPN1 were shown to be involved in regulation of Wnt-signaling^38,39^, a pathway that is dysregulated in MM^40^. The predicted miR-365 targets PIK3R3 and VGLL4 have both previously been validated as functional targets^41,42^.

## Conclusions

Taken together, we describe, for the first time, a comprehensive global analysis of miRNA-target interaction in MM. Sequencing-based expression analysis and correlation of miRNA and mRNA in primary MM cells enabled identification of novel functional targeting pairs, as confirmed by validation experiments. Thus, our sequencing study can be used by other researchers as a starting point or support for exploiting new miRNA interactions in MM. We identified miRNA target genes that are shown to be involved in cancer progression. It would therefore also be interesting to further investigate if any of the validated miRNA-mRNA target pairs play a role in MM pathogenesis.

## Methods

### Patient samples

CD138 + plasma cells were isolated from bone marrow aspirates obtained at diagnosis from 86 MM patients (Biobank 1, St. Olavs University Hospital HR, Trondheim, Norway). The cells were isolated using RoboSep automated cell separator and Human CD138 Positive Selection Kit (StemCell Technologies, Grenoble, France)^43^. The Regional Committee for Medical and Health Research Ethics (REK2011/2029) approved the study, and all patients provided written informed consent.

### RNA isolation, library preparation and sequencing

RNA for sRNA-seq and mRNA-seq was isolated from the same cell-pellet using miRVana total RNA isolation (ThermoFisher, #AM1560). Small RNA-seq libraries were randomly prepared from 400 ng of RNA using the NEXTFLEX Small RNA-Seq Kit v3 (PerkinElmer, #NOVA-5132-05) using 16 PCR cycles. 10 synthetic calibrator RNAs^44^ were mixed with the input RNA during the first ligation step. mRNA-seq libraries were randomly prepared using the TruSeq Stranded mRNA Library Prep Kit (Illumina # RS-122-2101) with 400 ng input RNA. The sequencing libraries were sequenced on the NextSeq 500 System from Illumina.

### Gene ontology analyses

Gene ontology analyses were performed using *clusterProfiler* in R. Expressed genes in the dataset were used as background. The p-values are indicated with colour and are adjusted for multiple testing using Benjamini Hochberg correction. “GeneRatio” is defined as k/n, where k = overlap of the input gene-list with the specific gene-set and n = overlap of the input gene-list with all the members of the collection of gene-sets. “Count” is the number of genes detected in the enrichment that belong to the specific GO-term. The filtering used for the GO analysis for predicted miRNA targets by miRDB and TargetScan was the 5% best targets for miRDB (miRDB value > 92.8) and TargetScan context score less than −0.366. In addition, we required the targets to have a negative correlation of less than −0.1.

### Transfection experiment

For the transfection- RNA-sequencing experiment, 2.5 × 10^6^ INA-6 MM cells were transfected in biological triplicates using 10 μM miRNA mimics and negative control miRNA. The mimics and negative control were purchased from miRIDIAN with catalog numbers: hsa-miR-125b-5p: #: C-300595-03-0005; has-miR-365b-3p: # C-301901-00-0005; microRNA Mimic Negative Control #1 Catalog ID:CN-001000-01-05. Transfected cells were incubated for 48 h before harvesting RNA using the miRVana total RNA isolation (ThermoFisher, #AM1561). The transfection for the qPCR experiment was performed similarly on two biological replicates followed by RT-qPCR using a StepOne Real-Time PCR System with the Taqman probes: H2AFY (Hs01016650_m1), VGLL4 (Hs00893985_m1), PIK3R3 (Hs01103591_m), EPN1 (Hs00203391_m1), MAN1B1 (Hs00359915_m1), MKNK2 (Hs00179671_m1), NEDD9 (Hs00610590_m1), RNF44 (Hs01556065_g1), and ACTB (Hs01060665_g1) as an endogenous control. MM cell line INA-6 was a kind gift from Dr. Martin Gramatzki (University of Erlangen-Nuremberg, Erlangen, Germany).

### Data processing small RNA-seq

The raw sequencing data was processed as previously described^44^, in addition to removing the random nucleotides associated with the NEXTFLEX small RNA library preparation kit.

### Data processing messenger RNA-seq

The raw sequencing data was quality-controlled using *fastQC*^45^. The reads were trimmed using fastq_quality filter with the parameters -Q33 -q 20 -p 80. The trimmed reads were mapped to the human genome (GRCh38.p7) using star aligner^46^ using default parameters. Reads were counted using *htseq-count* from the HTseq python package using the RefSeq GFF matching the genome version.

### Differential expression analysis

Differentially expressed mRNAs for the transfection experiment were detected using the *limma-voom* procedure in R^47^. Genes that were expressed with at least 1 count in more than 25% of the samples were included in the analysis. Genes with benjamini-hochberg-adjusted p-values less than 0.05 were regarded as significant. Ensembl IDs were converted to gene-symbols and entrez IDs using the *biomaRt* and *org.Hs.eg.db* packages in R.

We used the following procedure in *limma-voom* to detect differentially expressed genes (exemplified for miR-125):

- *require(limma)*
- *df.dge <-DGEList(df) #df is the input count matrix*
- *keep <-rowSums(df.dge$counts > 1) >  = dim(df.dge)[2]/4*
- *df.dge <-df.dge[keep,]*
- *group <-colnames(df)*
- *df.dge <-calcNormFactors(df.dge, method = "TMM")*
- *des <-model.matrix(~ 0 + group)*
- *v <-voom(df.dge,plot = T)*
- *fit <-lmFit(v, design = des)*
- *contrasts <-makeContrasts(miR125 = miR125-miRNEG,levels = des)*
- *fit2 <-contrasts.fit(fit, contrasts = contrasts)*
- *fit2 <-eBayes(fit2)*
- *colSums(decideTests(fit2)! = 0)*
- *topTable_miR_125 <-topTable(fit2,coef = "miR125",sort.by = "P",adjust.method = "BH", n = Inf)*

### Correlation and target analyses

Correlations between miRNAs and mRNAs were calculated in R using Pearson’s correlation and the function *cor*. Correlation p-values were calculated using the function *cor.mtest* from the R-pacakge *corrplot* (v0.84) and adjusted for multiple testing using the function *p.adjust* with Benjamini–hochberg correction. MiRNA targets were identified using TargetScanHuman (v.7.2)^2^ and miRDB^16^. For analysis on miR-125b and miR-365b we applied a miRDB filter of 85. Functionally validated miRNA targets were identified using miRTarBase (v2020)^48^. For the general target analysis (Fig. 1D) we included the top 40% highest expressed mRNAs, TargetScan targets with a context score less than −0.035 and the 5% best miRDB targets. The cumulative distribution-plots were generated in R using *stat_ecdf*, and the* p*-values between the groups were calculated using the Kolmogorov–Smirnov.

### Ethics approval and consent to participate

All patient samples were donated after informed consent, and the study approved by the Regional Committee for Medical Research Ethics Central Norway, REK Central (REC 2011/2029 and 2012/2033). The study was performed in accordance with the Helsinki Declaration.

### Consent for publication

There is no individual person’s data.

## Supplementary Information

Supplementary Information 1.

Supplementary Information 2.

Supplementary Information 3.

Supplementary Information 4.

## Data Availability

Due to Norwegian law on sensitive data, raw data cannot be submitted to public repositories, however, all raw data are available upon request to the corresponding author. Norwegian data protection is governed by the Law on the Processing of Personal Data (Personal Data Act) of 15 June 2018 (only available in Norwegian: https://lovdata.no/dokument/NL/lov/2018-06-15-38) ('the Act'), which implements the General Data Protection Regulation (Regulation (EU) 2016/679) ('GDPR'). Under this law, RNA sequencing data is interpreted to be data that directly or indirectly can identify a physical individual. miRNA and mRNA expression pairs and correlation clinical data are available through an interactive web application, on which additional associations can be investigated (https://github.com/MjelleLab/MicroRNA-and-Gene-Expression-In-Multiple-Myeloma). The processed count matrices for all RNA-classes as well as the clinical data are available on the same site.

## References

[1] Bartel DP (2004). MicroRNAs: genomics, biogenesis, mechanism, and function. Cell.

[2] Friedman RC, Farh KK, Burge CB, Bartel DP (2009). Most mammalian mRNAs are conserved targets of microRNAs. Genome Res..

[3] Khan AA, Betel D, Miller ML, Sander C, Leslie CS, Marks DS (2009). Transfection of small RNAs globally perturbs gene regulation by endogenous microRNAs. Nat Biotechnol..

[4] Gutierrez NC, Sarasquete ME, Misiewicz-Krzeminska I, Delgado M, De Las RJ, Ticona FV (2010). Deregulation of microRNA expression in the different genetic subtypes of multiple myeloma and correlation with gene expression profiling. Leukemia.

[5] Wang N, Zheng J, Chen Z, Liu Y, Dura B, Kwak M (2019). Single-cell microRNA-mRNA co-sequencing reveals non-genetic heterogeneity and mechanisms of microRNA regulation. Nat Commun..

[6] Chen L, Li C, Zhang R, Gao X, Qu X, Zhao M (2011). miR-17-92 cluster microRNAs confers tumorigenicity in multiple myeloma. Cancer Lett..

[7] Di Martino MT, Gulla A, Cantafio ME, Lionetti M, Leone E, Amodio N (2013). In vitro and in vivo anti-tumor activity of miR-221/222 inhibitors in multiple myeloma. Oncotarget.

[8] Pichiorri F, Suh SS, Ladetto M, Kuehl M, Palumbo T, Drandi D (2008). MicroRNAs regulate critical genes associated with multiple myeloma pathogenesis. Proc Natl Acad Sci USA..

[9] Xu S, Cecilia Santini G, De Veirman K, Vande Broek I, Leleu X, De Becker A (2013). Upregulation of miR-135b is involved in the impaired osteogenic differentiation of mesenchymal stem cells derived from multiple myeloma patients. PLoS ONE.

[10] Sun CY, She XM, Qin Y, Chu ZB, Chen L, Ai LS (2013). miR-15a and miR-16 affect the angiogenesis of multiple myeloma by targeting VEGF. Carcinogenesis.

[11] Yang Y, Li F, Saha MN, Abdi J, Qiu L, Chang H (2015). miR-137 and miR-197 induce apoptosis and suppress tumorigenicity by targeting MCL-1 in multiple myeloma. Clin Cancer Res..

[12] Handa H, Murakami Y, Ishihara R, Kimura-Masuda K, Masuda Y (2019). The role and function of microRNA in the pathogenesis of multiple myeloma. Cancers.

[13] Katiyar A, Kaur G, Rani L, Jena L, Singh H, Kumar L (2021). Genome-wide identification of potential biomarkers in multiple myeloma using meta-analysis of mRNA and miRNA expression data. Sci Rep..

[14] Misiewicz-Krzeminska I, Krzeminski P, Corchete LA, Quwaider D, Rojas EA, Herrero AB, et al. Factors regulating microRNA expression and function in multiple myeloma. Noncoding RNA. 2019;5(1). 10.3390/ncrna5010009 PMC6468559 30654527

[15] Lewis BP, Burge CB, Bartel DP (2005). Conserved seed pairing, often flanked by adenosines, indicates that thousands of human genes are microRNA targets. Cell.

[16] Chen Y, Wang X (2020). miRDB: an online database for prediction of functional microRNA targets. Nucleic Acids Res..

[17] Jiang Y, Luan Y, He D, Chen G (2017). miR-125b expression affects tumor growth of multiple myeloma via targeting MKK7. Int J Clin Exp Pathol..

[18] Unno K, Zhou Y, Zimmerman T, Platanias LC, Wickrema A (2009). Identification of a novel microRNA cluster miR-193b-365 in multiple myeloma. Leuk Lymphoma..

[19] Peterson S, Thompson J, Ufkin M, Sathyanarayana P, Liaw L, Congdon CB (2014). Common features of microRNA target prediction tools. Front. Genet..

[20] Seckinger A, Meissner T, Moreaux J, Benes V, Hillengass J, Castoldi M (2015). miRNAs in multiple myeloma: a survival relevant complex regulator of gene expression. Oncotarget.

[21] Zadran S, Remacle F, Levine RD (2013). miRNA and mRNA cancer signatures determined by analysis of expression levels in large cohorts of patients. Proc Natl Acad Sci USA..

[22] Tan H, Huang S, Zhang Z, Qian X, Sun P, Zhou X (2019). Pan-cancer analysis on microRNA-associated gene activation. EBioMedicine.

[23] Vincenz L, Jager R, O'Dwyer M, Samali A (2013). Endoplasmic reticulum stress and the unfolded protein response: targeting the Achilles heel of multiple myeloma. Mol Cancer Ther..

[24] Obeng EA, Carlson LM, Gutman DM, Harrington WJ, Lee KP, Boise LH (2006). Proteasome inhibitors induce a terminal unfolded protein response in multiple myeloma cells. Blood.

[25] Baranowska K, Misund K, Starheim KK, Holien T, Johansson I, Darvekar S (2016). Hydroxychloroquine potentiates carfilzomib toxicity towards myeloma cells. Oncotarget.

[26] Vogl DT, Stadtmauer EA, Tan KS, Heitjan DF, Davis LE, Pontiggia L (2014). Combined autophagy and proteasome inhibition: a phase 1 trial of hydroxychloroquine and bortezomib in patients with relapsed/refractory myeloma. Autophagy.

[27] Farh KK, Grimson A, Jan C, Lewis BP, Johnston WK, Lim LP (2005). The widespread impact of mammalian MicroRNAs on mRNA repression and evolution. Science.

[28] Pyronnet S, Imataka H, Gingras A-C, Fukunaga R, Hunter T, Sonenberg N (1999). Human eukaryotic translation initiation factor 4G (eIF4G) recruits Mnk1 to phosphorylate eIF4E. EMBO J..

[29] Shi Y, Frost P, Hoang B, Yang Y, Bardeleben C, Gera J (2014). MNK1-induced eIF-4E phosphorylation in myeloma cells: a pathway mediating IL-6-induced expansion and expression of genes involved in metabolic and proteotoxic responses. PLoS ONE.

[30] Shi Y, Frost P, Hoang B, Yang Y, Fukunaga R, Gera J (2013). MNK kinases facilitate c-myc IRES activity in rapamycin-treated multiple myeloma cells. Oncogene.

[31] Wang J, Da C, Su Y, Song R, Bai Z (2021). MKNK2 enhances chemoresistance of ovarian cancer by suppressing autophagy via miR-125b. Biochem. Biophys. Res. Commun..

[32] Zhang Y, Yan LX, Wu QN, Du ZM, Chen J, Liao DZ (2011). miR-125b is methylated and functions as a tumor suppressor by regulating the ETS1 proto-oncogene in human invasive breast cancer. Cancer Res..

[33] Pan S, Cheng X, Chen H, Castro PD, Ittmann MM, Hutson AW (2013). ERManI is a target of miR-125b and promotes transformation phenotypes in hepatocellular carcinoma (HCC). PLoS ONE.

[34] Rambow F, Bechadergue A, Luciani F, Gros G, Domingues M, Bonaventure J (2016). Regulation of melanoma progression through the TCF4/miR-125b/NEDD9 cascade. J Invest Dermatol..

[35] Cheung R, Pizza G, Chabosseau P, Rolando D, Tomas A, Burgoyne T, et al. Glucose-dependent miR-125b is a negative regulator of β-cell function. bioRxiv. 2021:2021.05.17.444559. 10.2337/db21-0803 PMC9998846 35476777

[36] Sebastian R, Hosogane EK, Sun EG, Tran AD, Reinhold WC, Burkett S (2020). Epigenetic regulation of DNA repair pathway choice by MacroH2A1 splice variants ensures genome stability. Mol. Cell.

[37] Yoon C, Lu J, Ryeom SW, Simon MC, Yoon SS (2021). PIK3R3, part of the regulatory domain of PI3K, is upregulated in sarcoma stem-like cells and promotes invasion, migration, and chemotherapy resistance. Cell Death Dis..

[38] Chang B, Tessneer KL, McManus J, Liu X, Hahn S, Pasula S (2015). Epsin is required for Dishevelled stability and Wnt signalling activation in colon cancer development. Nat. Commun..

[39] Jiao S, Li C, Hao Q, Miao H, Zhang L, Li L (2017). VGLL4 targets a TCF4–TEAD4 complex to coregulate Wnt and Hippo signalling in colorectal cancer. Nat. Commun..

[40] van Andel H, Kocemba KA, Spaargaren M, Pals ST (2019). Aberrant Wnt signaling in multiple myeloma: molecular mechanisms and targeting options. Leukemia.

[41] Zhu Y, Zhao H, Rao M, Xu S (2017). MicroRNA-365 inhibits proliferation, migration and invasion of glioma by targeting PIK3R3. Oncol Rep..

[42] Liu WH, Lu JJ, Yu RK, Zhou L, Yu Q, Li DF (2021). LINC00641 regulates prostate cancer cell growth and apoptosis via the miR-365a-3p/VGLL4 axis. Eur Rev Med Pharmacol Sci..

[43] Zahoor M, Westhrin M, Aass KR, Moen SH, Misund K, Psonka-Antonczyk KM (2017). Hypoxia promotes IL-32 expression in myeloma cells, and high expression is associated with poor survival and bone loss. Blood Adv..

[44] Mjelle R, Sellaeg K, Saetrom P, Thommesen L, Sjursen W, Hofsli E (2017). Identification of metastasis-associated microRNAs in serum from rectal cancer patients. Oncotarget.

[45] Andrews S. FastQC: a quality control tool for high throughput sequence data. 2019 Available from: https://www.bioinformatics.babraham.ac.uk/projects/fastqc/.

[46] Dobin A, Davis CA, Schlesinger F, Drenkow J, Zaleski C, Jha S (2013). STAR: ultrafast universal RNA-seq aligner. Bioinformatics.

[47] Law CW, Chen Y, Shi W, Smyth GK (2014). voom: Precision weights unlock linear model analysis tools for RNA-seq read counts. Genome Biol..

[48] Huang HY, Lin YC, Li J, Huang KY, Shrestha S, Hong HC, et al. miRTarBase 2020: updates to the experimentally validated microRNA-target interaction database. Nucleic Acids Res. 2019. 10.1093/nar/gkz896 PMC7145596 31647101

