# Supplementary material for: Paired miRNA- and messenger RNA-sequencing identifies novel miRNA-mRNA interactions in multiple myeloma
Source: Sci Rep. 2022 Jul 15;12:12147. doi: 10.1038/s41598-022-16448-0 (PMC9287335; doi:10.1038/s41598-022-16448-0)
Supplement: Supplementary file 1 — Supplementary Information 1. [file 41598_2022_16448_MOESM1_ESM.pdf]

Additional File 1

Paired miRNA- and messenger RNA-sequencing identifies novel miRNA-mRNA interactions in multiple myeloma

Supplementary Fig. 1: A) The panel “Library size” shows the total library size. The panel “Alignments” shows the alignment-statistics, including reads that align at one position in the genome (SingleAligned); reads that align at multiple positions in the genome (MultiAligned); and reads that do not align to the human genome (NotAligned). The panel “Features” shows reads that overlap either the miRNA database miRBase, or the database of non-coding RNAs, RNACentral. The panel “RNAs” shows the absolute expression of the main RNA-classes detected. **B)** Shown is the relative expression of the main RNA-classes detected. **C)** A reprehensive bioanalyzer result for the miRNA libraries. Shown is the length of the adapter-ligated RNA fragments after small RNA sequencing library preparation. The “miRNA-peak” has a length of 151 nucleotides. The peaks longer than the miRNA-peak represent snoRNA, tRNAs and other non-coding RNAs.

Supplementary Fig. 2: A) Cumulative distributions of miRNA-mRNA correlation coefficients. Plotted in brown are the intergenic miRNA-mRNA pairs for which the miRNA is not located within the mRNA and plotted in blue are the intragenic miRNA-mRNA pairs for which the miRNA is located within the mRNA. The p-value represents the difference between the two distributions and is calculated using a one-sided Kolmogorov–Smirnov test. **B)** Top five positively correlated intragenic miRNA-mRNA pairs. The pairs are indicated in the facets of the plot, the miRNA expression is shown on the y-axis and the mRNA-expression is shown on the x-axis. **C)** Top five positively correlated intergenic miRNA-mRNA pairs.

Supplementary Fig. 3: Experimental setup for the miRNA transfection experiment.

Figure S1

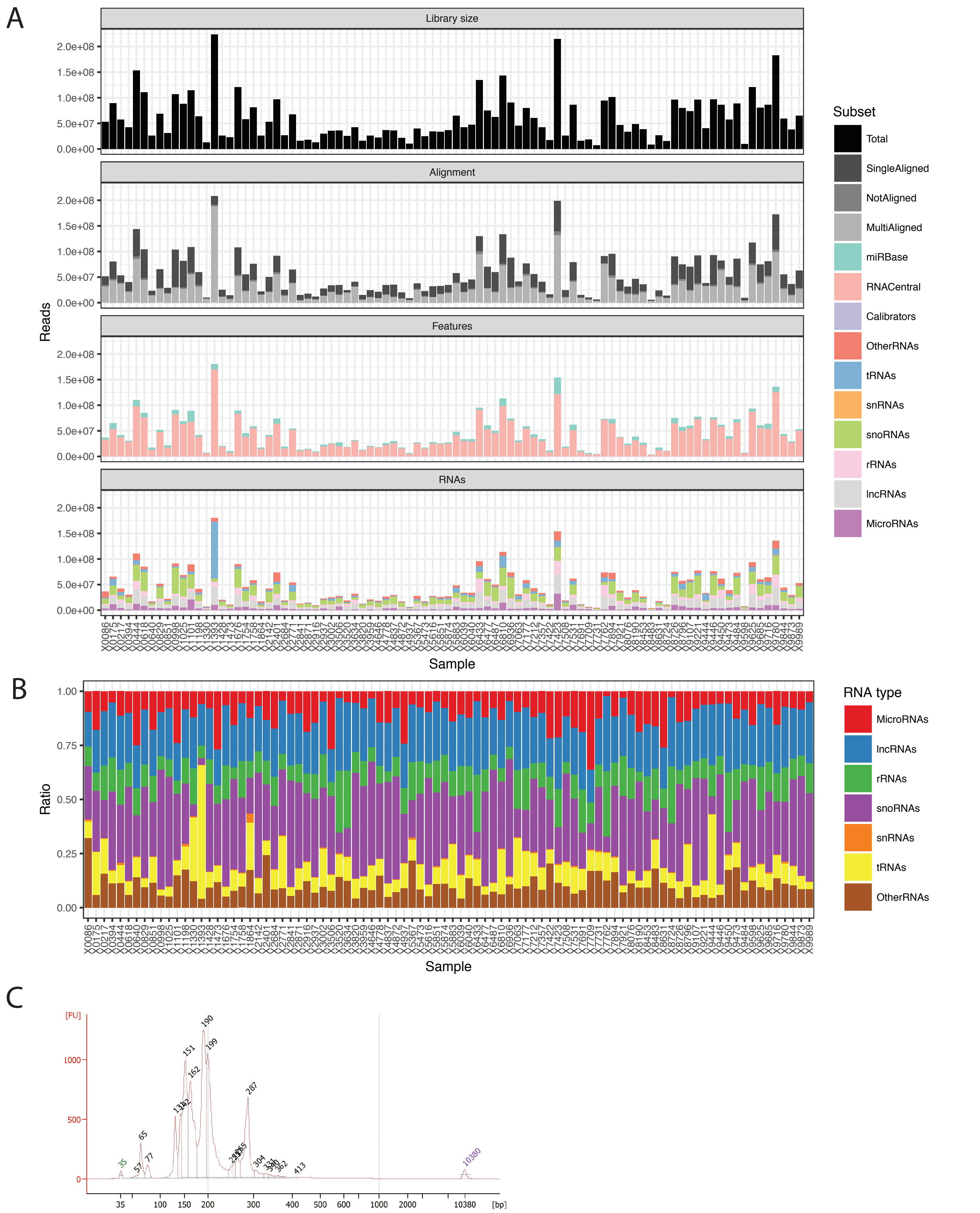

A
Library size
Alignment
Features
RNAs
Reads
Sample
0.0e+00
5.0e+07
1.0e+08
1.5e+08
2.0e+08
Subset
Total
SingleAligned
NotAligned
MultiAligned
miRBase
RNACentral
Calibrators
OtherRNAs
tRNAs
snRNAs
snoRNAs
rRNAs
lncRNAs
MicroRNAs
B
Ratio
Sample
0.00
0.25
0.50
0.75
1.00
RNA type
MicroRNAs
lncRNAs
rRNAs
snoRNAs
snRNAs
tRNAs
OtherRNAs
C
[FU]
1000
500
0
[bp]
35
100
150
200
300
400
500
600
1000
2000
10380

Figure S2

A

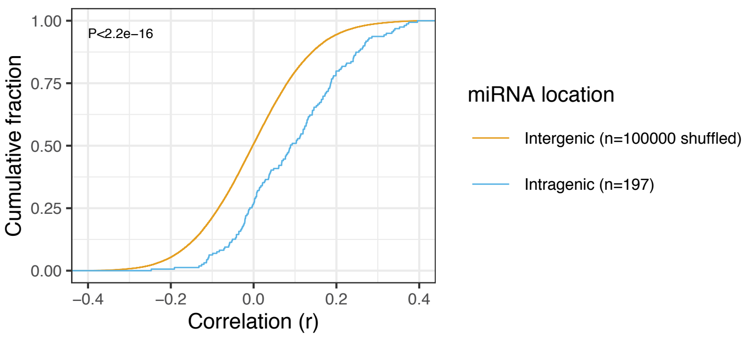

B

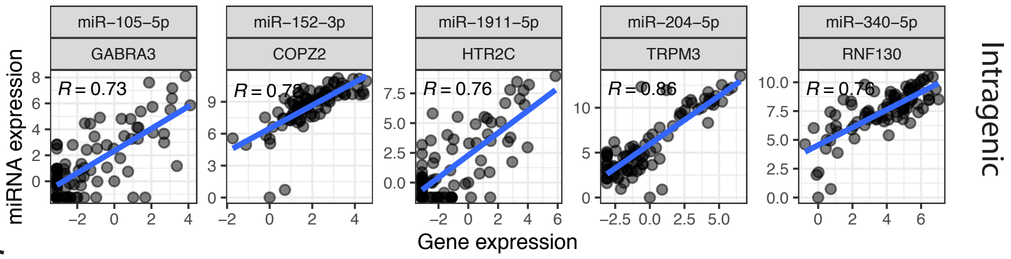

C

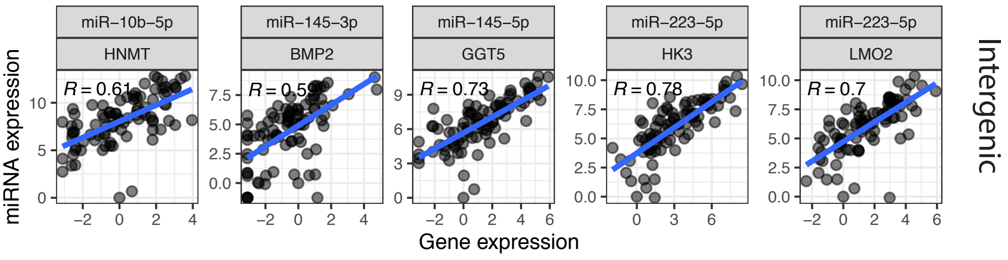

Figure S3

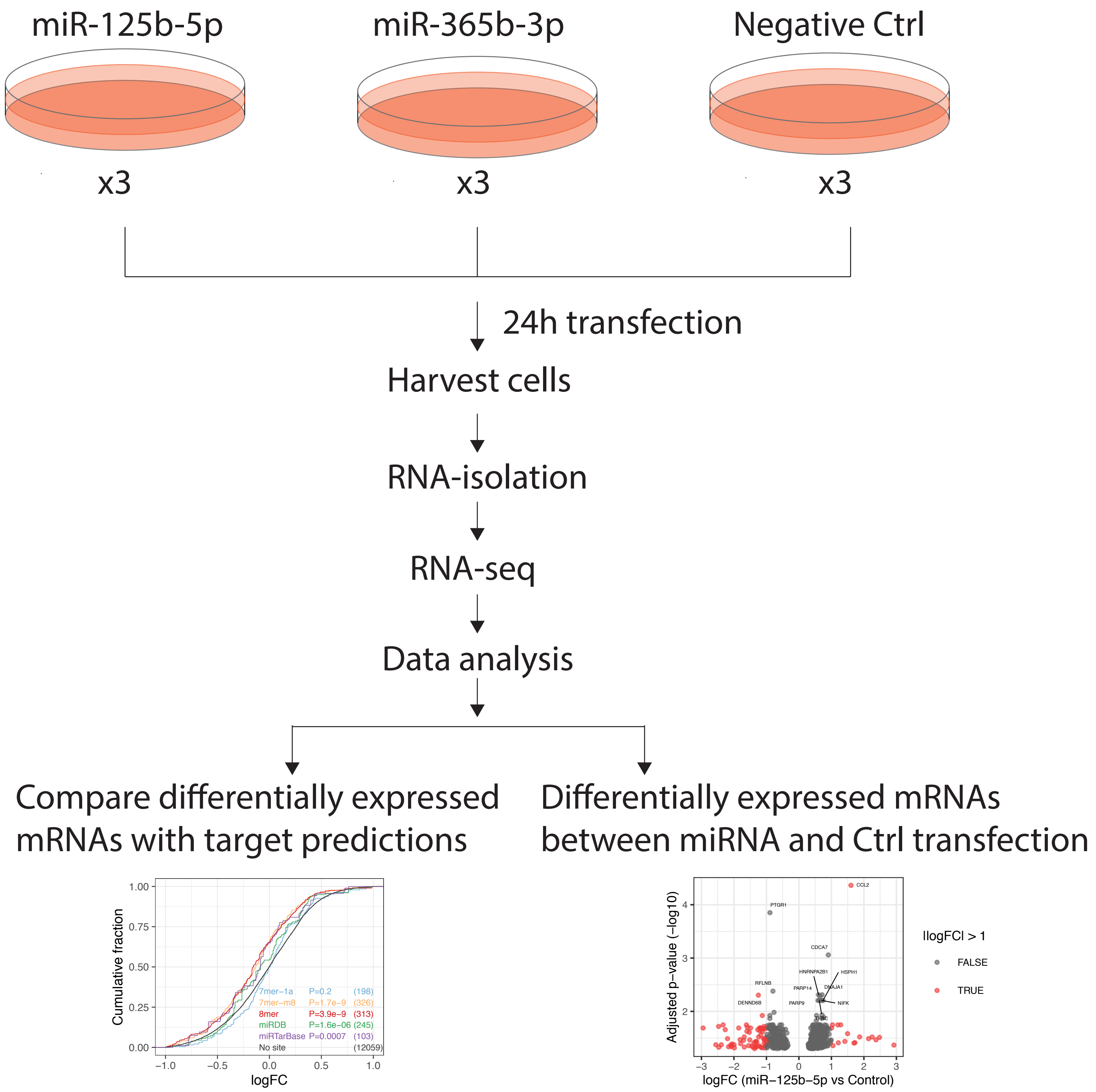
miR-125b-5p
miR-365b-3p
Negative Ctrl
x3
x3
x3
24h transfection
Harvest cells
RNA-isolation
RNA-seq
Data analysis
Compare differentially expressed mRNAs with target predictions
Differentially expressed mRNAs between miRNA and Ctrl transfection
Cumulative fraction
logFC
7mer−1a P=0.2 (198)
7mer−m8 P=1.7e−9 (326)
8mer P=3.9e−9 (313)
miRDB P=1.6e−06 (245)
miRTarBase P=0.0007 (103)
No site (12059)
Adjusted p−value (−log10)
logFC (miR−125b−5p vs Control)
|logFC| > 1
FALSE
TRUE
CCL2
PTGR1
CDCA7
HNRNPA2B1
HSPH1
RFLNB
PARP14
DNAJA1
DENND6B
PARP9
NIFK
